# A Vitamina D e o Aparelho Cardiovascular

**DOI:** 10.36660/abc.20240189

**Published:** 2024-06-03

**Authors:** Claudio Leinig Pereira da Cunha

**Affiliations:** 1 Universidade Federal do Paraná Clínica Médica Curitiba PR Brasil Universidade Federal do Paraná - Clínica Médica, Curitiba, PR – Brasil

**Keywords:** Vitamina D, Sistema Cardiovascular, Vitaminas Lipossolúveis, Calciferol

A vitamina D (ou calciferol) é uma vitamina solúvel em gordura. É um termo genérico e refere-se a um grupo de compostos lipossolúveis com uma estrutura de colesterol de quatro anéis. A síntese dérmica, sob ação da luz UV-B na pele, é a principal fonte natural desta vitamina, visto que muito poucos alimentos contêm vitamina D naturalmente (os peixes gordurosos, como salmão, atum, sardinha, truta, são a exceção).^1^ Esta vitamina D, proveniente da dieta ou da síntese dérmica, é biologicamente inativa. Apresenta-se como vitamina D3 (colecalciferol) e vitamina D2 (ergocalciferol), que são convertidas enzimaticamente no fígado em 25(OH)D (25-hidroxivitamina D = calcidiol), a principal forma circulante de vitamina D. Depois, no rim, o calcidiol é convertido em calcitriol (1,25-di-hidroxivitamina D), a forma ativa da vitamina D, que aumenta a absorção intestinal do cálcio, aumenta a reabsorção óssea e diminui a excreção renal do cálcio e fosfato.^2^

O melhor indicador laboratorial da adequação da vitamina D é a concentração sérica de 25(OH)D. No entanto não há consenso sobre a concentração ideal; a *National Academy of Medicine* indicou que 20 ng/ml é suficiente,^3^ mas a maioria dos especialistas ( *Endocrine Society, National Osteoporosis Foundation, International Osteoporosis Foundation, American Geriatrics Society* )^4^ sugerem que um nível mínimo de 30ng/ml seja necessário para os adultos.

A vitamina D e seus metabólitos têm reconhecido papel clínico na homeostase do cálcio e no metabolismo ósseo. A deficiência de vitamina D foi originalmente descoberta como a causa do raquitismo. Posteriormente, mostrou-se que a suplementação com vitamina D e cálcio diminuiu o risco de fraturas osteoporóticas em idosos.^5^

Além do seu papel metabólico relativo ao cálcio e aos ossos, a vitamina D contribui para a regulação de muitas outras funções celulares. O receptor de vitamina D (VDR) é quase universalmente expresso em células nucleadas, aproximadamente 3% do genoma humano está sob controle do calcitriol, que converte a vitamina D em sua forma ativa, e, portanto, o espectro de atividade do sistema endócrino da vitamina D é muito amplo. Entre os efeitos "extraesqueléticos" da vitamina D se destacam seu efeito na função muscular, no câncer e nos sistemas imunológico, metabólico e cardiovascular.^6^

Na edição dos Arquivos Brasileiros de Cardiologia, Faria et al.,^7^ estudam os efeitos da suplementação de vitamina D na hemodinâmica e no sistema nervoso autônomo de indivíduos obesos, com resultados favoráveis desta ação.^7^

Embora estudos observacionais mostrem uma associação entre baixo nível de vitamina D e maior risco de eventos cardiovasculares, não há consenso entre os ensaios randomizados que analisam o benefício da suplementação de vitamina D.^8^

Meta-análise de 19 estudos prospectivos, incluindo 65.994 pacientes, mostrou uma relação inversa entre os níveis de 25(OH)D (variando de 8 a 24 ng/ml) e o risco cardiovascular (risco relativo 1,03, IC 95% 1,00-1,60).^9^

Em outras revisões sistemáticas e meta-análises, entretanto, não houve efeito da suplementação de vitamina D nos desfechos cardiovasculares.^10 , 11^ As meta-análises também não mostraram um efeito significativo da vitamina D nos fatores de risco cardiovascular (pressão arterial, lipídios e glicose).^10^

Dados epidemiológicos em larga escala sobre populações predominantemente brancas na América do Norte e na Europa sugerem uma associação entre níveis baixos de 25(OH)D (<20 ng/ml) e alto risco de mortalidade por todas as causas. Em algumas meta-análises observou-se redução modesta da mortalidade, notadamente em pacientes mais velhos, não gravemente enfermos e com deficiência de vitamina D.^12^ Em ensaios com adultos gravemente doentes ou em adultos sem deficiência de vitamina D, não houve redução na mortalidade com a suplementação da vitamina D.^13^

Na análise da mortalidade cardiovascular, estudo prospectivo com dados do NHANES mostrou associação inversa entre níveis de 25(OH)D <21 ng/ml e mortalidade.^14^ Porém estes achados não têm se repetido em pesquisas que envolvem a suplementação de vitamina D em grande número de pacientes, buscando a redução da mortalidade cardiovascular.^15 , 16^

Desta forma, embora estudos observacionais indiquem uma associação entre a vitamina D e a saúde extraesquelética, inclusive a cardiovascular, faltam estudos randomizados com grande número de pacientes por tempo prolongado, que definam se a vitamina D pode oferecer benefícios preventivos e terapêuticos em ampla gama de alterações não-esqueléticas.
