# Supplementary material for: A Vitamina D e o Aparelho Cardiovascular
Source: Arq Bras Cardiol. 2024 Jun 3;121(5):e20240189. [Article in Portuguese] doi: 10.36660/abc.20240189 (PMC11164439; doi:10.36660/abc.20240189)
Supplement: Translation

# Translation: Vitamin D and the Cardiovascular System

**DOI:** 10.36660/abc.20240189i

**Authors:** Claudio Leinig Pereira da Cunha

**Affiliations:** 1 Curitiba PR Brazil Universidade Federal do Paraná - Clínica Médica, Curitiba, PR – Brazil

**Keywords:** Vitamin D, Cardiovascular System, Fat Soluble Vitamins, Cholecalciferol

Vitamin D (or calciferol) is a fat-soluble vitamin. It is a generic term and refers to a group of fat-soluble compounds with a four-ring cholesterol structure. Dermal synthesis, under the action of UV-B light on the skin, is the main natural source of this vitamin, as very few foods contain vitamin D naturally (fatty fish, such as salmon, tuna, sardines, trout, are the exception).^1^This vitamin D, whether derived from the diet or dermal synthesis, is biologically inactive. It is presented as vitamin D3 (cholecalciferol) and vitamin D2 (ergocalciferol), which are enzymatically converted in the liver into 25(OH)D (25-hydroxyvitamin D = calcidiol), the main circulating form of vitamin D. Then, in the kidney, calcidiol is converted into calcitriol (1,25-dihydroxyvitamin D), the active form of vitamin D, which increases intestinal absorption of calcium, increases bone reabsorption and decreases renal excretion of calcium and phosphate.^2^

The best laboratory indicator of vitamin D adequacy is the serum 25(OH)D concentration. However, there is no consensus on the ideal concentration; the National Academy of Medicine indicated that 20 ng/ml is suficiente,^3^but most experts (Endocrine Society, National Osteoporosis Foundation, International Osteoporosis Foundation, American Geriatrics Society)^4^suggest that a minimum level of 30ng/ml is necessary for adults.

Vitamin D and its metabolites have a recognized clinical role in calcium homeostasis and bone metabolism. Vitamin D deficiency was originally discovered as the cause of rickets. Subsequently, supplementation with vitamin D and calcium was shown to decrease the risk of osteoporotic fractures in the elderly.^5^

In addition to its metabolic role in calcium and bones, vitamin D contributes to the regulation of many other cellular functions. The vitamin D receptor (VDR) is almost universally expressed in nucleated cells, approximately 3% of the human genome is under the control of calcitriol, which converts vitamin D to its active form, and therefore the spectrum of activity of the endocrine system of vitamin D is very broad. Among the "extraskeletal" effects of vitamin D, its effect on muscle function, cancer, and the immune, metabolic, and cardiovascular systems stands out.^6^

In this edition of *Arquivos Brasileiros de Cardiologia* , Faria et al.^7^study the effects of vitamin D supplementation on hemodynamics and the autonomic nervous system of obese individuals, with favorable results from this action.^7^

Although observational studies show an association between low vitamin D levels and increased risk of cardiovascular events, there is no consensus among randomized trials analyzing the benefit of vitamin D supplementation.^8^

Meta-analysis of 19 prospective studies, including 65,994 patients, showed an inverse relationship between 25(OH)D levels (ranging from 8 to 24 ng/ml) and cardiovascular risk (relative risk 1.03, 95% CI 1.00-1.60).^9^

In other systematic reviews and meta-analyses, however, there was no effect of vitamin D supplementation on cardiovascular outcomes.^10 , 11^Meta-analyses also did not show a significant effect of vitamin D on cardiovascular risk factors (blood pressure, lipids, and glucose).^10^

Large-scale epidemiological data on predominantly white populations in North America and Europe suggest an association between low 25(OH)D levels (<20ng/ml) and a high risk of mortality from all causes. In some meta-analyses, a modest reduction in mortality was observed, notably in older, non-seriously ill patients with vitamin D deficiency.^12^In trials with seriously ill adults or in adults without vitamin D deficiency, there was no reduction in mortality. with vitamin D supplementation.^13^

In the analysis of cardiovascular mortality, a prospective study with data from the NHANES showed an inverse association between 25(OH)D levels <21 ng/ml and mortality.^14^However, these findings have not been repeated in research involving vitamin D supplementation in a large number of patients, seeking to reduce cardiovascular mortality.^15 , 16^

Thus, although observational studies indicate an association between vitamin D and extraskeletal health, including cardiovascular, there is a lack of randomized studies with a large number of patients over a long period, that define whether vitamin D can offer preventive and therapeutic benefits in a wide range of non-skeletal disorders.
